# The Nuclear Receptor Nr4a1 Controls CD8 T Cell Development Through Transcriptional Suppression of Runx3

**DOI:** 10.1038/srep09059

**Published:** 2015-03-12

**Authors:** Heba N. Nowyhed, Tridu R. Huynh, Amy Blatchley, Runpei Wu, Graham D. Thomas, Catherine C. Hedrick

**Affiliations:** 1 Division of Inflammation Biology, La Jolla Institute for Allergy and Immunology, La Jolla, CA

## Abstract

The NR4A nuclear receptor family member Nr4a1 is strongly induced in thymocytes undergoing selection, and has been shown to control the development of T_reg_ cells; however the role of Nr4a1 in CD8^+^ T cells remains undefined. Here we report a novel role for Nr4a1 in regulating the development and frequency of CD8^+^ T cells through direct transcriptional control of *Runx3*. We discovered that Nr4a1 recruits the corepressor, CoREST to suppress *Runx3* expression in CD8^+^ T cells. Loss of Nr4a1 results in increased *Runx3* expression in thymocytes which consequently causes a 2-fold increase in the frequency and total number of intrathymic and peripheral CD8^+^ T cells. Our findings establish Nr4a1 as a novel and critical player in the regulation of CD8 T cell development through the direct suppression of *Runx3*.

Nr4a1 (Nur77), Nr4a2 (Nurr1) and Nr4a3 (NOR-1) constitute the NR4A subfamily of orphan nuclear receptors within the steroid thyroid receptor family. These nuclear receptors function as transcription factors to either induce or repress gene transcription^1^. In the thymus, Nr4a1 is activated as part of the immediate early response downstream of TCR signaling^2^,^3^, and serves as an indicator for strength of TCR signals^4^,^5^. Nr4a1 has been previously described to play a role in the apoptosis of CD4^+^CD8^+^ (DP) thymocytes bearing αβ T-cell receptors (TCRs) with a high affinity for self-antigens in the thymus^6^,^7^. Dominant negative and constitutively-active mutants of Nr4a1 resulted in decreased and increased negative selection respectively^7^,^8^, implicating Nr4a1 as an important transcription factor in the negative selection of thymocytes^9^.

Members of the NR4A family have been reported to both positively and negatively regulate pro-inflammatory genes^10^. Previous studies demonstrate a potent anti-inflammatory activity of Nurr1 in microglia and astrocytes mediated by a Nurr1/CoREST transrepression pathway^11^. Furthermore, it was found that overexpression of Nur77 and Nor1 in a macrophage cell line can suppress iNOS activation in response to LPS, suggesting that the CoREST transrepression pathway may be widely used by members of the NR4A family^11^.

The current perspective on lineage commitment to CD4 versus CD8 SP cells is that commitment results from the opposing activity of two transcriptional repressors, Thpok/cKrox (encoded by *Zbtb7b*) and Runx3. Thpok is critical for CD4 commitment while Runx3 is critical for commitment to the CD8 lineage^12^. Regulation of CD4 and CD8 expression is critical for the appropriate selection of the TCRαβ repertoire during thymocyte differentiation. Despite the apparent symmetry of this process, there is evidence for Thpok having a dominant effect on Runx3, as Thpok antagonizes Runx3 functions in cells where both factors are expressed. *Runx3* has been shown to be up-regulated during the differentiation of CD4^+^CD8^+^(DP) thymocytes into CD8 cells in the thymus^13^. Runx factors have been shown to not only be involved in CD4 silencing but to also directly affect positive transcriptional control of *Cd8*^14^.

Based on the analysis of thymocytes lacking *Runx3*, Taniuchi et. al. proposed that *Runx3* is involved in the epigenetic silencing of the *Cd4* gene at the CD8 SP (single positive) stage^13^. Through the use of Runx3 transgenic mice, overexpression of transgenic Runx3 specifically within the T cell lineage resulted in a large increase in the CD4^−^CD8^+^ thymocyte population^15^.

In this report we examined the role of Nr4a1 in CD8^+^ T cells. We show that Nr4a1 intrinsically controls CD8^+^ T cell development and frequency through the regulation of *Runx3* expression by binding the distal promoter region of Runx3 and suppressing *Runx3* expression in developing CD8^+^ T cells through interaction with CoREST. In the absence of Nr4a1, this suppression of Runx3 is lost, resulting in an increase of Runx3 expression and consequently a 2-fold increase in the number and frequency of intrathymic and peripheral CD8^+^ T cells. We confirmed these findings in human T cells by finding that Nr4a1 interacts with CoREST and suppresses Runx3 expression in human CD8^+^ T cells upon αCD3/CD28 stimulation. Thus, our data in mouse and human CD8^+^ T cells identify Nr4a1 as a key transcriptional repressor that controls Runx3 expression, regulating the development of CD8^+^ T cells.

## Results

### Nr4a1 regulates thymic and peripheral CD8^+^ T cell frequency

**Figure 1:**
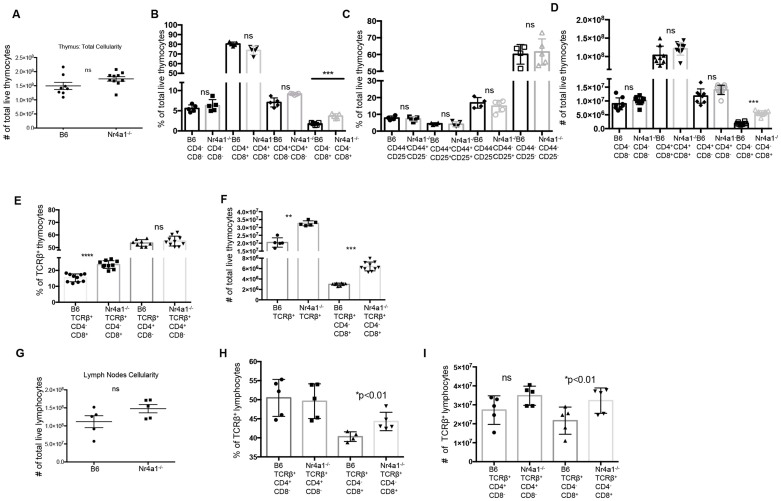
Nr4a1 regulates thymic and peripheral CD8^+^ T cell frequency. (A) Total live cell count represented as total cellularity of the thymi of B6 (wildtype Nr4a1-intact mice) versus Nr4a1*^−/−^* mice. (B) Frequency of CD4^−^CD8^−^, CD4^+^CD8^+^, CD4^+^CD8^−^, and CD4^−^CD8^+^ of total live thymocytes in B6 versus Nr4a1*^−/−^* mice. (C) Frequency of CD44^+^CD25^−^, CD44^+^CD25^+^, CD44^−^CD25^+^, CD44^−^CD25^−^ of total live CD4^−^CD8^−^ thymocytes. (D) Total live cell number of CD4^−^CD8^−^, CD4^+^CD8^+^, CD4^+^CD8^−^, and CD4^−^CD8^+^ of total live thymocytes in B6 versus Nr4a1*^−/−^* mice. (E) Frequency of CD4^+^CD8^−^, and CD4^−^CD8^+^ of total live TCRβ^+^ thymocytes. (F) Total live cell number of TCRβ^+^ thymocytes, and TCRβ^+^ CD4^−^CD8^+^ thymocytes. (G) Total live cell count represented as total cellularity of the lymph nodes of B6 versus Nr4a1*^−/−^* mice. (H) Frequency of CD4^+^CD8^−^, and CD4^−^CD8^+^ of total live TCRβ^+^ lymphocytes. (I) Total live cell number of CD4^+^CD8^−^, and CD4^−^CD8^+^ of total live TCRβ^+^ lymphocytes. Data are representative of three separate experiments with at least three age and sex matched mice per group. Each symbol represents an individual mouse; small horizontal lines indicate the mean. *P* value, unpaired, two-tailed *t*-test. *p < 0.01, **p < 0.001, ***p < 0.0001.

To study the role of Nr4a1 in CD8^+^ T cells we utilized *Nr4a1*-deficient mice on a congenic C57BL/6J background. Nr4a1*^−/−^* mice showed no significant differences in total cell numbers in the thymus and lymph nodes (Figs 1a,g). We analyzed T cell populations within the thymus and found no significant differences in the frequency and total number of CD4^−^CD8^− ^(DN), CD4^+^CD8^+^(DP), and CD4^+^CD8^−^ cells (Figs 1b,d and Supplementary Fig 1a). We further analyzed the frequency of the four stages within the DN population and also found no significant differences (Fig 1c and Supplementary Fig 1b). However, the CD8^+^CD4^− ^population within the thymus was increased by 2-fold, from an average of 1.5% of the total population in B6 mice to 3.5% in Nr4a1*^−/−^* mice (Fig 1b). The total number of CD8^+^CD4^−^ cells also increased from an average of 1.8 × 10^6^ cells in B6 mice to 6 × 10^6^ cells in Nr4a1*^−/−^* mice (Fig 1d). Analysis of cells within the thymus based solely on CD4 and CD8 expression will include both mature TCRβ^+^ cells and immature cells that have not completed TCRαβ rearrangement and expression. Therefore we analyzed the TCRβ^+^CD4^+^CD8^−^ and TCRβ^+^CD8^+^CD4^−^ T cell populations and found no significant differences in the frequency of mature CD4^+ ^T cells, but found a 2-fold increase in the mature CD8^+^ T cell population in Nr4a1*^−/−^*thymus (Fig 1e and Supplementary Figs 1c,d). The total number of TCRβ^+^CD8^+^CD4^−^ cells was also increased from an average of 3 × 10^6^ cells in B6 mice to 6.8 × 10^6^ cells in Nr4a1*^−/−^* mice (Fig 1f), which accounted for the increase of total number of TCRβ^+ ^cells (Fig 1f).

Lymph node cellularity was not significantly different in Nr4a1-deficient mice (Fig 1g). We analyzed the T cell population in the lymph nodes and found no change in the TCRβ^+^CD4^+^population, however the frequency and total number of TCRβ^+^CD8^+ ^was also significantly increased in the absence of Nr4a1 (Figs 1h,I and Supplementary Figs 1g,h). The absence of Nr4a1 appears to specifically impact the frequency and total number of CD8^+^ T cells in both the thymus and the periphery, thus we asked if different levels of Nr4a1 expression impacts CD8^+^ T cell frequency. To do so we analyzed Nr4a1^+/− ^mice. The mRNA level of *Nr4a1* in the heterozygous mice is half that of Nr4a1^+/+^ mice (Supplemental Fig 2a), therefore these mice are a useful tool to study how different levels of Nr4a1 expression impacts T cell development/frequency. A significant negative linear correlation was found between Nr4a1 expression and the frequency of TCRβ^+^CD8^+^CD4^−^ T cells found in the thymus and lymph nodes (Supplemental Figs 2b,c).

### Nr4a1 controls CD8 T cell development through *Runx3* expression

We set out to decipher if the higher frequency and total number of CD8^+^ T cells within the thymus of Nr4a1-deficient mice was a direct result of an aberrancy in CD8^+^ T cell development. We first examined the rate of proliferation of thymocytes in B6 versus Nr4a1-deficient mice through analysis of BrdU incorporation. Total thymocytes, CD4^+^CD8^+^ (DP), total TCRβ^+^, TCRβ^+^CD4^+^CD8^−^, and TCRβ^+^CD8^+^CD4^−^ cells all showed no differences in proliferation (Supplemental Fig 3a), therefore the increase in frequency and total number of CD8^+^ T cells is not due to an increase in the proliferation of developing thymocytes. Previous reports have found that a transgenic dominant-negative Nur77 protein can inhibit the apoptotic process that accompanies negative selection of thymocytes; however, Nr4a1-deficient mice should have no such phenotype due to the redundancy of Nr4a1 and Nr4a3 in T cell apoptosis^4^,^16^. To confirm, we analyzed the frequency of apoptotic cells in the CD4^+^CD8^+^ (DP), total TCRβ^+^, TCRβ^+^CD4^+^CD8^−^, and TCRβ^+^CD8^+^CD4^− ^populations and indeed confirmed that Nr4a1 deficiency does not affect apoptosis of developing thymocytes (Supplementary Fig 3b).

CD24 (HSA) is highly expressed on developing thymocytes and is down regulated upon emigration to the periphery^17^. To confirm that the increase in CD8^+^ T cells found in the thymus is not due to recirculation of peripheral CD8^+^ T cells, we analyzed CD24 expression on CD8^+^CD4^−^ and CD4^+^CD8^− ^thymocytes and lymphocytes from B6 and Nr4a1*^−/−^* mice. CD8^+^CD4^−^ and CD4^+^CD8^− ^isolated from the thymus of both B6 and Nr4a1*^−/−^* mice highly expressed CD24 while those isolated from the lymph nodes had down regulated CD24, indicative of peripheral T cells (Supplementary Fig 4a). These data suggest that the T cells within the thymus of Nr4a1*^−/−^*mice are developing in the thymus and not recirculating peripheral T cells. To further confirm that the increase of intrathymic CD8^+ ^T cells in Nr4a1*^−/−^* mice was not due to peripheral CD8 T cells preferentially homing to the thymus, purified B6 or *Nr4a1^−/−^* CD8^+^ T cells isolated from the lymph nodes were labeled with Carboxyfluorescein succinimidyl ester (CFSE) and intravenously injected into B6 or *Nr4a1^−/−^* mice. The ability of the transferred cells to migrate back to the thymus was assessed 10 days post transfer (Supplementary Fig 4b). Although the transferred *Nr4a1^−/−^* and B6 CFSE labeled CD8 T cells were easily detectable in the lymph nodes of the recipient mice, no transferred cells could be found in the thymi of the host mice.

**Figure 2:**
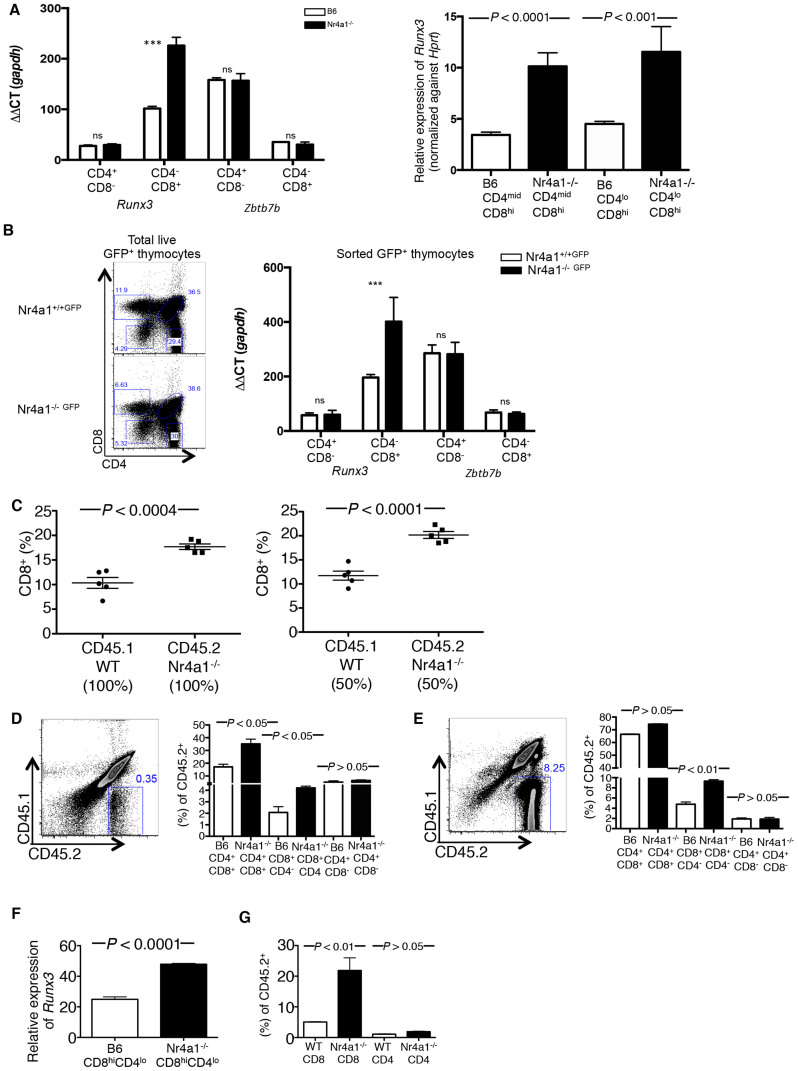
Nr4a1 intrinsically controls CD8^+^ T cell development through Runx3 expression. (A) qPCR measuring the levels of *Runx3 and Zbtb7b* RNA relative to *Gapdh* from sorted CD4^+^CD8^− ^and CD4^−^CD8^+^ cells isolated from thymi of B6 versus Nr4a1*^−/−^* mice. qPCR measuring the levels of *Runx3* RNA relative to *Hprt* from sorted CD4^mid^CD8^hi ^and CD4^lo^CD8^hi^ cells isolated from thymi of B6 versus Nr4a1*^−/−^* mice. (B) qPCR measuring the levels of *Runx3 and Zbtb7b* RNA relative to *Gapdh* from sorted GFP^+^CD4^+^CD8^−^ and GFP^+^CD4^−^CD8^+^ thymocytes from Nr4a1^+/+GFP+^ versus Nr4a1*^−/−^*^GFP+^ mice. (C) B6, Nr4a1*^−/−^*, and B6:Nr4a1*^−/−^*1:1 mixed bone marrow chimeric mice were analyzed for the frequency of TCRβ^+^CD8^+^ Each symbol represents an individual mouse; small horizontal lines indicate the mean. (D) Isloated CD45.2 Common Lymphoid Progenitor (CLP) cells from B6 or Nr4a1*^−/−^* bone marrow were adoptively transferred into sublethally irradiated CD45.1.2 B6 hosts. Frequency of CD45.2^+^(left plot) DP(CD4^+^CD8^+^), TCRβ^+^ CD8^+^, and TCRβ^+^ CD4^+^ T cells (right graph) 7 days after transfer and (E) 14 days after transfer. (F) qPCR measuring *Runx3* RNA levels in CD45.2 CD8^hi^CD4^lo^ T cells isolated from the thymus 14 days after transfer. (G) Frequency of CD45.2 TCRβ^+^ CD8^+^, and TCRβ^+^ CD4^+^ T cells from peripheral lymph nodes 14 days after transfer. Data are representative of three separate experiments with at least three age and sex matched mice per group. Small horizontal lines indicate the mean. *P* value, unpaired, two-tailed *t*-test. ns(p > 0.05), *p < 0.01, **p < 0.001, ***p < 0.0001.

The zinc finger protein Thpok (also called *Zbtb7b*) is necessary for the generation of CD4 T cells, whereas the transcription factor Runx3 is important for CD8 T cell development^14^,^18^,^19^. Kohu *et al.* previously reported that overexpression of Runx3 increases the proportion and total number of CD8^+^ T cells and that Runx3 possesses the capacity to actively drive thymocytes toward the CD8 lineage^15^. We sorted CD8^+^CD4^−^ and CD4^+^CD8^−^ populations from thymus of B6 and Nr4a1*^−/−^* mice and analyzed mRNA levels of *Runx3* and *Zbtb7b* by qPCR (Fig 2a). We found a significant increase in *Runx3* expression in the CD8^+^CD4^− ^population of Nr4a1-deficient mice, whereas Thpok levels were normal in the CD4^+^CD8^− ^population in Nr4a1-/- mice. Moreover, CD4^mid^CD8^hi^ and CD4^lo^CD8^hi^ thymocytes sorted from Nr4a1*^−/−^*mice (Fig 2a and Supplementary Fig 5a) also showed significantly higher levels or *Runx3* compared to the same populations from B6 mice, suggesting that Nr4a1 may function in thymocytes to suppress *Runx3*.

To further explore this hypothesis, we analyzed the proportion of CD8^+^CD4^−^ and CD4^+^CD8^−^ cells in TCRβ^lo^CD24^hi ^(immature) and TCRβ^hi^CD24^mid ^(mature) thymic populations as previous studies have reported that overexpression of *Runx3* in developing thymocytes causes an increase in both immature and mature CD8^+^CD4^− ^cells^15^,^20^ In support of our hypothesis that *Runx3* expression is increased in Nr4a1*^−/−^* mice, we found an increase in the frequency of CD8^+^CD4^−^ cells in both immature and mature thymic populations in the absence of Nr4a1 (Supplemental Fig 4c). We further confirmed our finding by analyzing CD69^+^ thymocytes (a marker for positive selection) expressing high levels of TCRβ (mature) and medium degree of TCRβ expression (premature) (Supplemental Fig. 4d). In concordance to our previous results, CD8^+^CD4^−^ cells were significantly increased in both TCRβ^mid^CD69^hi ^and TCRβ^hi^CD69^hi^ populations in Nr4a1*^−/−^* mice.

*Runx3* regulates expression of genes associated with CTL differentiation and function, specifically, *Gzmb*, *Ifng, Eomes*, and perforin (*Prf1*)^21^,^22^. To test if increased *Runx3* expression in Nr4a1-deficient thymocytes functionally drove the up-regulation of *Runx3* target genes, we sorted CD8^+^CD4^−^ and CD4^+^CD8^−^ cells from B6 and Nr4a1*^−/−^* thymi and analyzed mRNA levels of *Ifng, Eomes, Gzmb,* and *Prf1* (Supplemental Fig 4e). The mRNA for all 4 genes were significantly elevated in CD8^+^CD4^−^ isolated from Nr4a1*^−/−^* mice while no significant differences were observed in mRNA expression levels within CD4^+^CD8^−^ cells. Thus, the increased Runx3 in CD8^+^ T cells in the absence of Nr4a1 is functional and influences the expression of CTL associated genes.

### Nr4a1 functions in a cell intrinsic manner to regulate CD8 T cell development and *Runx3* expression

Through the use of Nr4a1^GFP^ mice, studies have shown that Nr4a1 is up-regulated in lymphocytes by antigen receptor stimulation but not by inflammatory stimuli. Furthermore, it has been established that Nr4a1 increases in CD8 T cells undergoing positive selection and lineage commitment^5^. We crossed Nr4a1^GFP^ with Nr4a1*^−/−^* mice to generate Nr4a1^GFP/−^. These mice are functional Nr4a1 knockouts that report GFP expression in cells that would normally express Nr4a1. We generated these mice to track Nr4a1.GFP expression in the Nr4a1-deficient mice. We sorted GFP^+^CD4^+^CD8^−^ and GFP^+^CD8^+^CD4^− ^from Nr4a1^GFP/−^ and Nr4a1^GFP/+^ thymi and analyzed *Runx3* and Thpok mRNA levels by qPCR (Fig 2b). We found a significant increase in the expression level of *Runx3* in GFP^+^CD8^+^CD4^− ^cells isolated from Nr4a1^GFP/−^ mice, further supporting that Nr4a1 expression is necessary for proper regulation of Runx3 expression.

To ask whether Nr4a1 functions in a cell intrinsic manner in CD8^+ ^T cells, we utilized a mixed chimera approach. Chimeric mice were established by engrafting B6 hosts with an equal (50:50) mix of *Nr4a1^−/−^* and B6 bone marrow cells. Control chimeric mice were also generated where 100% *Nr4a1^−/−^* or B6 bone marrow cells were transplanted into B6 hosts (Figs 2c and Supplemental Figs 5b,c,d). Analysis of chimeras 10 weeks post transplant revealed that B6 TCRβ^+^CD8^+^ thymocytes developed at a normal frequency while Nr4a1-deficient TCRβ^+^CD8^+^ thymocytes developed at a significantly higher frequency (Fig. 2c). As expected, B6 and Nr4a1-deficient TCRβ^+^CD4^+^ cells developed normally with no significant difference seen in the frequency of the CD4^+^ T cells (Supplemental Fig 2d).

To further confirm a cell-intrinsic effect we isolated common lymphoid progenitors (CLP) from the bone marrow of CD45.2^+^ Nr4a1*^−/−^* and CD45.1^+^ B6 mice using a no-touch magnetic bead based negative selection kit. The CLPs from either the Nr4a1-deficient or B6 bone marrow were adoptively transferred into sub-lethally irradiated CD45.1.2^+^ B6 hosts. We analyzed the developing thymocyte population 7 and 14 days post transfer (Figs 2d,e). Through this chimera transfer system we were able to analyze early CD8 and CD4 T cell development in transferred Nr4a1*^−/−^*CLPs in the setting of a B6 thymus in that over 90% of all cells found within the host thymus were Nr4a1-intact B6 cells. We found under such conditions that only the transferred Nr4a1*^−/−^* cells produced a higher frequency of CD8^+^CD4^−^ T cells within the first 7 days (Fig 2d), and the frequency of CD4^+^CD8^−^ cells remained normal. After 14 days of transfer, the differences in CD8^+^CD4^−^ T cell frequency became even greater within the thymus (Fig 2e) and a significant increase in peripheral Nr4a1 deficient TCRβ^+^CD8^+^ T cells was also found at this time point (Fig 2g). We then isolated B6 CD45.1^+^CD8^+^CD4^−^ and Nr4a1*^−/−^*CD45.2^+^CD8^+^CD4^−^ T cells from the thymus 14 days after transfer and analyzed *Runx3* mRNA expression by qPCR (Fig 2f). We found *Runx3* expression to be just over 2-fold higher in the absence of Nr4a1. Therefore, our data show that Nr4a1 functions intrinsically within CD8 cells to regulate *Runx3* expression and their development.

### Nr4a1 directly suppresses Runx3

**Figure 3:**
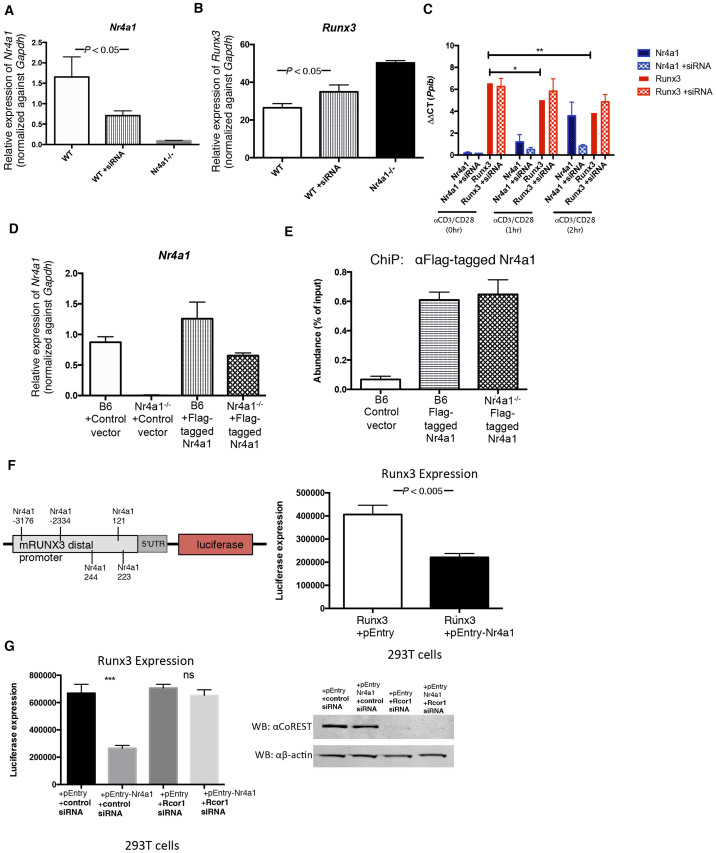
Nr4a1 directly controls induction of Runx3. (A) siRNA knockdown of Nr4a1 in αCD3,CD28 activated TCRβ^+^ CD8^+^ lymphocytes and qPCR analysis of *Nr4a1* expression upon knockdown. (B) qPCR measuring the levels of *Runx3* RNA relative to the *Gapdh* in αCD3,CD28 activated TCRβ^+^ CD8^+^ lymphocytes from B6, B6 treated with siRNA Nr4a1 knockdown, and Nr4a1*^−/−^*mice. (C) qPCR measuring the levels of *Nr4a1* and *Runx3* RNA relative to *Ppib* in human CD8^+^ T cells. CD8^+^ T cells were negatively isolated from whole human blood and *in vitro* activated with αCD3,CD28 for 0, 1, and 2 hrs. “+siRNA” indicates samples treated with siRNA against *Nr4a1.* (D) Transfection of Flag-tagged Nr4a1 into B6 and Nr4a1*^−/−^*αCD3,CD28 activated TCRβ^+^ CD8^+^ lymphocytes and qPCR analysis of *Nr4a1* RNA relative to the *Gapdh* expression upon transfection. (E) Chromatin-immunoprecipitation of Flag-tagged Nr4a1 at the distal *Runx3* promoter in αCD3,CD28 activated TCRβ^+^ CD8^+^ lymphocytes. Cells were transfected with a control or Flag-tagged Nr4a1. (F) Luciferase promoter assay was performed with 293T cells. The cells were transfected with either the promoter region of Runx3 and either an empty vector or the Nr4a1 open reading frame. Nearly 18 hrs post transfection lucerifase activity was measured. (G) Luciferase promoter assay was performed with 293T cells. The cells were first transfected with either the non targeting siRNA or Rcor1 siRNA. 36 hrs later the promoter region of Runx3 and either an empty vector or the Nr4a1 open reading frame were transfected. Nearly 18 hrs post transfection lucerifase activity was measured. Knockdown of CoREST in the treated 293T cells was confirmed by western blot using an antibody against CoREST. Data are representative of two separate experiments with at least three age and sex matched mice per group. Small horizontal lines indicate the mean. *P* value, unpaired, two-tailed *t*-test. ns(p > 0.05), *p < 0.01, **p < 0.001, ***p < 0.0001.

We next investigated if Nr4a1 directly regulates *Runx3* expression. We first asked how *Runx3* expression would be affected upon reducing *Nr4a1* levels by using siRNA approaches. We isolated mature CD8^+^ T cells from peripheral lymph nodes of B6 mice, and activated the CD8 T cells with αCD3CD28 over the course of siRNA administration. After 72hrs we found close to 70% reduction in *Nr4a1* expression (Fig 3a). We analyzed the siRNA-treated cells by qPCR and found a corresponding 30-40% increase in *Runx3* (Fig 3b). We also isolated CD8^+^ T cells from human blood donor samples and activated the CD8^+^ T cells with αCD3αCD28 for 0,1, and 2hrs and analyzed *Nr4a1* and *Runx3* levels by qPCR (Fig 3c). As expected, *Nr4a1* levels rose after 1 and 2hrs of αCD3CD28 stimulation. *Runx3* levels significantly dropped upon stimulation, suggesting that Nr4a1 functions to suppress Runx3 in human CD8 T cells. To examine this possibility, we reduced *Nr4a1* expression in human CD8 T cells using siRNA approaches. We administered siRNAs against *Nr4a1* for 48 hours prior to adding αCD3CD28 for 0,1, and 2 hrs (Fig 3c). *Nr4a1* levels were reduced by over 80% in the samples treated with siRNA, with no significant reduction of *Runx3* after 1 hr and 2 hrs of αCD3αCD28 stimulation, further suggesting that Nr4a1 functions in human CD8^+^ T cells as a repressor of Runx3.

Therefore, Nr4a1 expression negatively correlates with Runx3 levels, suggesting that Nr4a1 may control Runx3 expression by directly binding to the Runx3 promoter. The Runx3 distal promoter contains five putative binding sites for Nr4a1 (−121, −223, −244, −2334, −3176). To confirm that Nr4a1 directly regulates Runx3, we performed chromatin immunoprecipitation (ChiP) for Nr4a1 at the Runx3 distal promoter. We transfected both B6 and Nr4a1*^−/−^* CD8^+^ T cells isolated from peripheral lymph nodes with Flag-tagged Nr4a1 (Fig 3d). Upon ChiP analysis we detected Nr4a1 on the *Runx3* distal promoter (Fig 3e) clearly demonstrating that Nr4a1 directly controls the expression of *Runx3*.

To further test the repressive function of Nr4a1 on Runx3 transcription, we performed a luciferase promoter-reporter assay in 293T cells. We transfected 293Ts with the promoter region of Runx3 (1–3973) along with either an empty vector or a vector containing the Nr4a1 open reading frame (Fig 3F). We find that Nr4a1 transfection reduces *Runx3* promoter-reporter induction, indicating that Nr4a1 functions to repress *Runx3* gene induction by binding to the *Runx3* promoter region. Previous reports have demonstrated that Nr4a receptor family can function to inhibit gene expression by direct binding to the gene promoter and subsequent recruitment of the CoREST corepressor complex^11^,^23^. To test if Nr4a1 collaborates with CoREST to directly repress the expression of Runx3, we setup the same luciferase promoter-reporter assay in 293T cells that was performed in Figure 3F. We further treated the 293T cells with either a non-targeting control siRNA or siRNA against Rcor1 (CoREST) (Fig 3G). We confirmed knockdown of CoREST by western blot (Fig 3G, right panel). Upon knockdown of CoREST, Nr4a1 repression of Runx3 was lost; therefore our data clearly demonstrates that Nr4a1 functions to directly repress Runx3 expression through recruitment of the CoREST corepressor complex.

### Nr4a1 binds to CoREST to directly suppress Runx3

**Figure 4:**
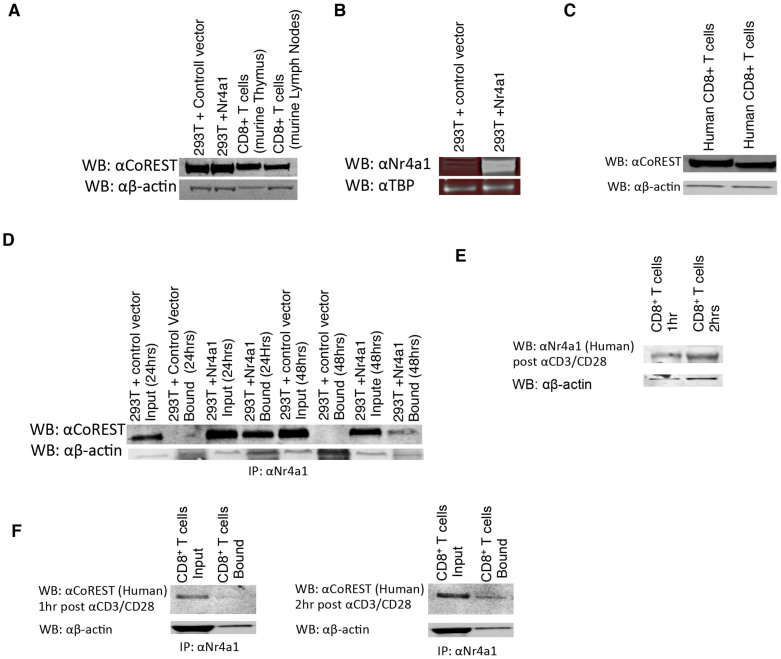
Nr4a1 directly binds to CoREST (A) Western Blot analyses of CoREST expression in 293T cells transfected with an open reading frame, 293T cells transfected with Nr4a1, TCRβ^+^CD8^+^ T cells isolated from B6 thymi, and TCRβ^+^CD8^+^ T cells isolated from B6 lymph nodes. (B) Western Blot analyses of Nr4a1 expression in 293T cells transfected with a control vector, and 293T cells transfected with Nr4a1. (C) Western Blot analyses of CoREST expression from two different samples of CD8^+^ T cells negatively isolated from whole human blood. (D) Analyses of the interaction of Nr4a1 and CoREST. Co-IP was performed with anti-Nr4a1 antibody and western blots were developed with anti-CoREST antibody. (E) Western Blot analyses of Nr4a1 expression in CD8^+^ T cells isolated from human blood and stimulated *in vitro* with αCD3αCD28 dynabeads for 1 hr and 2 hrs. (F) Analyses of the interaction of Nr4a1 and CoREST in human CD8^+^ T cells isolated from the blood and stimulated *in vitro* with αCD3αCD28 dynabeads for 1 hr and 2 hrs. Co-IP was performed with anti-Nr4a1 antibody and western blots were developed with anti-CoREST antibody. Data are representative of two separate experiments. (A–F) Blots shown have been cropped from full length blot. Full length blots are shown in Supplemental Figure 6a–f. Supplemental Fig. 6a corresponds to Figure 4a, Supplmental Fig. 6b corresponds to Figure 4b, etc.

We next set out to test if Nr4a1 directly binds to CoREST. We first verified by western blot analysis the presence of CoREST in CD8^+ ^T cells isolated from the thymus and lymph nodes of B6 mice (Fig 4a) as well as CD8^+^ T cells isolated from human donor blood (Fig 4c). Saijo *et al.* reported that Nurr1 binds to CoREST and the Nurr1/CoREST complex mediates suppression of promoter activity^11^. We asked if Nr4a1 directly binds to CoREST to form a Nr4a1/CoREST complex that functions to suppress Runx3. We performed a Co-IP on 293T cells transfected with the Runx3 distal promoter region and either a control vector or Nr4a1. Samples were collected 24 and 48 hrs after transfection. We immuno-precipitated Nr4a1 and performed western blot for CoREST (Fig 4d). Nr4a1 clearly binds to CoREST within 24 hrs of transfection and remains bound after 48 hrs. We also performed Co-IP on human CD8^+^ T cells isolated from blood, and stimulated the cells with αCD3αCD28 dynabeads for either 1 or 2 hrs. Our previous qPCR analysis demonstrated upregulation of *Nr4a1* after αCD3/CD28 stimulation (Fig 3c). We confirmed up-regulation of Nr4a1 in human cells upon CD3/CD28 stimulation by western blot (Fig 4e). We immuno-precipitated Nr4a1 and performed western blot analysis for CoREST on human samples stimulated for both 1 and 2 hrs (Fig 4f). Our data clearly demonstrates that Nr4a1 binds to CoREST in both human and mouse CD8^+^ T cells.

## Discussion

While much work has been done in understanding the function of Nr4a1 in CD4 T cells, its role in CD8 T cells remains poorly understood. Here we have discovered a novel role for Nr4a1 in regulating the development of CD8 T cells through the direct transcriptional suppression of *Runx3*. We found that Nr4a1 functions intrinsically within CD8 T cells and we clearly demonstrate that our observed phenotype is not due to the extrinsic effect of bystander cell populations within the thymus. Transcriptional programs can function in a dose dependent manner; through analysis of Nr4a1^+/−^ mice we found a linear, negative correlation between Nr4a1 and the frequencies of both thymic and peripheral CD8 T cells.

Recent studies have found that Nr4a2 represses anti-inflammatory genes by recruiting the CoREST corepressor complex^11^. These investigators also determined that Nr4a1 and Nr4a3 exhibit similar suppressive roles, thereby concluding that the CoREST transrepression pathway is widely used by members of the NR4A family. Through ChiP analysis, we have found that mechanistically Nr4a1 suppresses Runx3 by directly binding to the distal promoter region of the transcription factor. Nr4a1 recruits and binds to CoREST, utilizing the CoREST transrepression pathway to suppress Runx3 transcription.

The absence of Nr4a1 results in a unique impact on CD8 T cell development and frequency as the CD4 T cell population remains at a normal frequency and absolute number. CD4-committed cells express high levels of ThPOK, a strong inhibitor of *Runx3*. If the absence of Nr4a1 causes a rise in *Runx3* within the CD4 committed cells, ThPOK expression would outcompete Runx3 and abrogate an effect on CD4 T cell development. The levels of ThPOK were not different in CD4 T cells isolated from the thymus of B6 and Nr4a1-deficient mice (Figs 2a,b). Therefore, we have identified a novel role for Nr4a1 in the suppression of *Runx3* and thereby, the specific regulation of CD8^+^ T cell development.

We expanded our studies to include human CD8 T cells. Nr4a1 is known to be expressed in human PBMCs^1^,^10^. We found that Nr4a1 levels rise in CD8 T cells isolated from human blood upon αCD3/CD28 stimulation after 1 and 2 hrs (Figs 3c, 4e). Through siRNA approaches, we demonstrated that, parallel to our observations in murine CD8^+^ T cells, Nr4a1 negatively regulates Runx3 (Fig 3c). We went on to show that in stimulated human CD8^+^ T cells, Nr4a1 binds to CoREST (Fig 4f). Thus, Nr4a1 binds to CoREST and can function to suppress Runx3 expression in circulating human CD8 T cells. Thus, selective manipulation of Nr4a1 expression may serve as a potential strategy to boost CD8 T cell response against infection and during vaccination through its manipulation of Runx3.

## Methods

### Animals

C57BL/6J wild-type mice (000664), B6.SJL-*Ptprc^a^ Pepc^b^*/BoyJ (002014) CD45.1 mice, and *Nr4a1*^−/−^ mice on a congenic C57BL/6J background (006187) were from The Jackson Laboratory. Mice were fed a standard rodent chow diet and were housed in microisolator cages in a pathogen-free facility. The NR4A1-GFP reporter mice were generated as described, were a kind gift of Dr Kristin A. Hogquist (University of Minnesota), and are now available from The Jackson Laboratory (016617). All experiments followed guidelines of the La Jolla Institute for Allergy and Immunology Animal Care and Use Committee, and approval for use of rodents was obtained from the La Jolla Institute for Allergy and Immunology according to criteria outlined in the Guide for the Care and Use of Laboratory Animals from the National Institutes of Health. Mice were euthanized by CO_2 _inhalation.

### Flow Cytometry and Antibodies

Thymi, and lymph nodes were excised and pushed through a 70-μm strainer, and bone marrow cells from both femurs and tibias were collected by centrifugation. All samples were collected in Dulbecco's PBS (Gibco) and were stored on ice during staining and analysis. Red blood cells were lysed in RBC Lysis Buffer according to the manufacturer's protocol (BioLegend). Cells (2 × 10^6^ to 4 × 10^6^) were resuspended in 100 μl flow staining buffer (1% BSA (wt/vol) and 0.1% (wt/vol) sodium azide in PBS). Fcγ receptors were blocked for 15 min and surface antigens on cells were stained for 30 min at 4°C. LIVE/DEAD Fixable Dead Cell Stain (Invitrogen) was used for analysis of viability, and forward- and side-scatter parameters were used for exclusion of doublets from analysis. For intracellular cytokine staining, cells were stimulated for 2 h with phorbol myristate acetate (50 ng/ml) and ionomycin (1 g/ml; Sigma-Aldrich) in the presence of brefeldin A (GolgiPlug; BD Biosciences).For additional intracellular staining, cells were fixed and made permeable with the Cytofix/Cytoperm Fixation/Permeabilization Solution Kit (BD Biosciences). Cells were stained for 30 min at 4°C with directly conjugated fluorescent antibodies. The absolute number of cells was calculated by multiplication of the percentage of live cells in individual subsets by the total cell count before staining. Calculations of percentages were based on live cells as determined by forward and side scatter and viability analysis. Cell fluorescence was assessed with a FACSCalibur (BD Biosciences) and was analyzed with FlowJo software (version 9.2). Mean fluorescence intensity was quantified, and expression was calculated relative to that of the wild-type control. For staining of thymocytes from NR4A1-GFP mice, thymi were collected and prepared as previously described and 5 × 10^6^ cells were incubated for 30 min at 4°C in 30 μl flow staining buffer (1% (wt/vol) BSA and 0.1% (wt/vol) sodium azide in PBS) with the appropriate antibodies in the presence of Fc Block (BD Biosciences). Cellular fluorescence was assessed with an LSR II, FACSAria II or FACSCalibur (BD Biosciences) and data were analyzed with FlowJo software (TreeStar), CD69-PercP, CD25-PE, CD44-alexa flour 700, CD4-APC, CD4-FITC, CD8-PE Texas Red, CD8- PercpCy5.5, TCRbeta-APC eFlour 780, were commercially purchased from either eBioscience or BD Pharmingen.

### Adoptive transfers

CD8^+^ T cells were isolated with an EasySep Mouse CD8 Enrichment kit according to the manufacturer's instructions (Stem Cell Technologies). CD8 cells were labeled by incubation for 10 min at 37°C with 2.5 μM CFSE (5,6-carboxyfluorescein diacetate succinimidyl ester; Molecular Probes). 2 × 10^7^ cells were injected i.v. in 200μl PBS into unirradiated recipient mice. Quantitative real-time PCR. Thymocyte cell populations were isolated by flow cytometry and total cellular RNA was collected with an RNeasy Plus Micro Kit according to the manufacturer's protocol (Qiagen). RNA purity and quantity was measured with a nanodrop spectrophotometer (Thermo Scientific). Approximately 500 ng RNA was used for synthesis of cDNA with an Iscript cDNA Synthesis Kit (Bio-Rad). Total cDNA was diluted 1:20 in H_2_O, and a volume of 9 μl was used for each real-time condition with a MyIQ Single-Color Real- Time PCR Detection System (Bio-Rad) and TaqMan Gene Expression Mastermix and TaqMan primers. Data were analyzed and presented on the basis of the relative expression method. The formula for this calculation was as follows: relative expression = 2^−(SΔCT–CΔCT)^, where ΔC_T _is the change in cycling threshold between the gene of interest and the ‘housekeeping’ gene *Gapdh* (encoding glyceraldehyde phosphate dehydrogenase), S is the result obtained with *Nr4a1*^−/−^ cells, and C is the result obtained with C57BL/6J control cells. The expression of each wild-type transcript was calculated relative to the mean wild-type transcript expression to show variability in wild-type samples. The mean wild-type transcript expression was then compared with that of transcripts from each *Nr4a1*^−/−^ sample to determine the change relative to wild-type expression.

### Bone marrow chimera studies

Recipient mice (wild-type or *Nr4a1*^−/−^) were irradiated in two doses of 550 rads each (for a total of 1,100 rads) 4 h apart. Bone marrow cells from both femurs and tibias of donor mice (wild-type or *Nr4a1*^−/−^) were collected under sterile conditions. Approximately 5 × 10^6^ nucleated bone marrow cells were obtained from each donor mouse. Bones were centrifuged for the collection of marrow, then cells were washed and resuspended in Dulbecco's PBS for injection. Approximately 5 × 10^6^ unfractionated bone marrow cells in 200 μl media were delivered retro-orbitally into each recipient mouse. Recipient mice were housed in a barrier facility under pathogen- free conditions before and after bone marrow transplantation. After bone marrow transplantation, mice were provided autoclaved acidified water with antibiotics (trimethoprim-sulfamethoxazole) and were fed autoclaved food. Mice were used for experiments after 6 weeks of bone marrow reconstitution. Wild-type CD45.1 and CD45.2 in *Nr4a1*^−/−^ and C57BL/6 mice (wild-type CD45.2) were used for tracking cells in chimeras that received mixed–bone marrow transplantation. For this transplantation, 2.5 × 10^6 ^cells from *Nr4a1*^−/−^ mice (CD45.2^+^) and 2.5 × 10^6 ^cells from B6.CD45.1 mice were mixed at a ratio of 1:1 for reconstitution of recipients (wild-type CD45.1^+^ or wild-type CD45.2^+^) as described above.

### Transfection of plasmids

Cells were treated with plasmid or siRNA complexes using Lipofectamine 2000 (Invitrogen) in Opti-MEM media (Invitrogen) at 37°C overnight following manufacturer's instructions. The specific silencing of target genes was confirmed by qRT-PCR

### Chromatin immunoprecipitation assay

(2 × 10^7^) CD8^+^ T-cells isolated from peripheral lymph nodes using a CD8^+ ^selection kit (EasySep) were activated with plate bound anti-CD3, CD28 overnight. CD8^+ ^cells were then transfected with Flag-tagged Nr4a1 and cross-linked with 2 mM DSG (Sigma) and 1% formaldehyde methanol-free (Thermo Scientific Pierce). Cells were then lysed in RIPA buffer with protease inhibitors (Sigma), and chromatin was sheared by sonication in a Bioruptor sonicator (Diagenode). Immunoprecipitation was carried out with Dynabeads Protein A (Life Technologies) and Anti-Flag antibody (Origene). DNA was isolated using a ChIP DNA Clean and Concentrator column (Zymo). Target genes were amplified from the isolated DNA using SYBR® green master mixture (Roche). anti-Nur77 (M-210) and Anti-CoREST (Santa Cruz) was used for IP.

### Luciferase assay

Motifs were cloned into PCMV-6 vector (Promega). Luciferase constructs were transfected into 293T cells. The following day, 14–18 hours later, cells were lysed and luciferase assays were performed using a Dual-Luciferase Reporter Assay System (Promega) on a single automatic injection Mithras (Berthold technologies) luminometer following the manufacturer's protocol. Transfection of each construct was performed in triplicate in each assay. Empty vector was transfected in each plate in triplicate in both conditions to be used for normalization purposes. Luciferase readings were taken as singlets. Ratios of Renilla luciferase readings to Firefly luciferase readings were taken for each experiment and triplicates were averaged. The average values of the tested constructs were normalized to the activity of the empty construct.

## Author Contributions

H.N. and C.H. wrote the manuscript text. H.N. prepared figures 1–4 and all supplemental figures. T.H. assisted with figures 3–4. R.W. assisted with figure 4. A.B. assisted with figure 3. G.T. assisted with figure 3. All authors reviewed the manuscript.

## Supplementary Material

Supplementary Information Supplemental Figures and Legends
