# Supplementary material for: The Nuclear Receptor Nr4a1 Controls CD8 T Cell Development Through Transcriptional Suppression of Runx3
Source: Sci Rep. 2015 Mar 12;5:9059. doi: 10.1038/srep09059 (PMC4356985; doi:10.1038/srep09059)
Supplement: Supplementary Information — Supplemental Figures and Legends [file srep09059-s1.pdf]

Supplemental Figure 1

The nuclear receptor Nr4a1 controls CD8 T cell development through transcriptional control of Runx3.

Heba N. Nowyhed, Tridu R. Huynh, Amy Blatchley, Runpei Wu, Graham D. Thomas and Catherine C. Hedrick

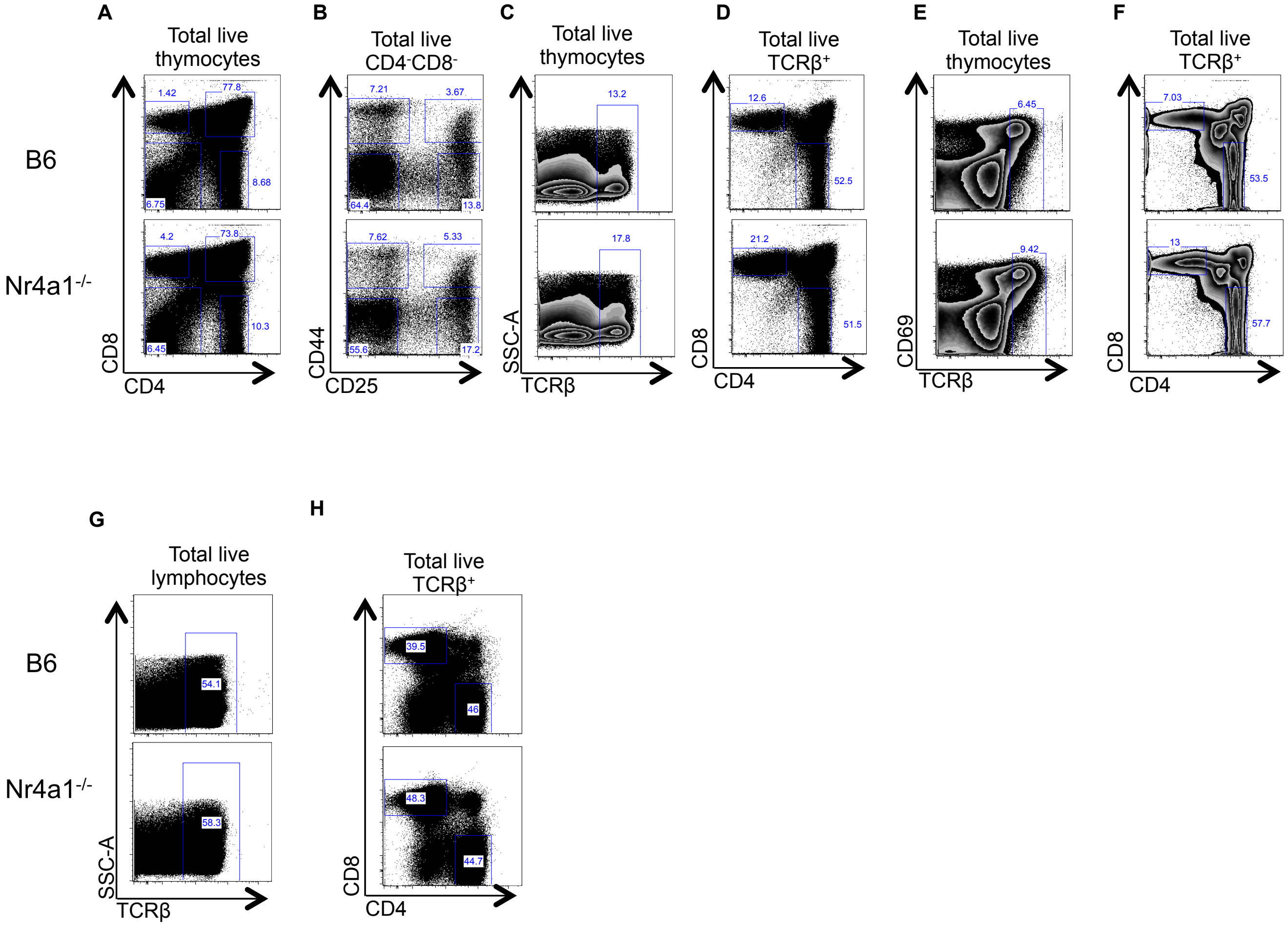

Supplemental Figure 2

The nuclear receptor Nr4a1 controls CD8 T cell development through transcriptional control of Runx3.

Heba N. Nowyhed, Tridu R. Huynh, Amy Blatchley, Runpei Wu, Graham D. Thomas and Catherine C. Hedrick

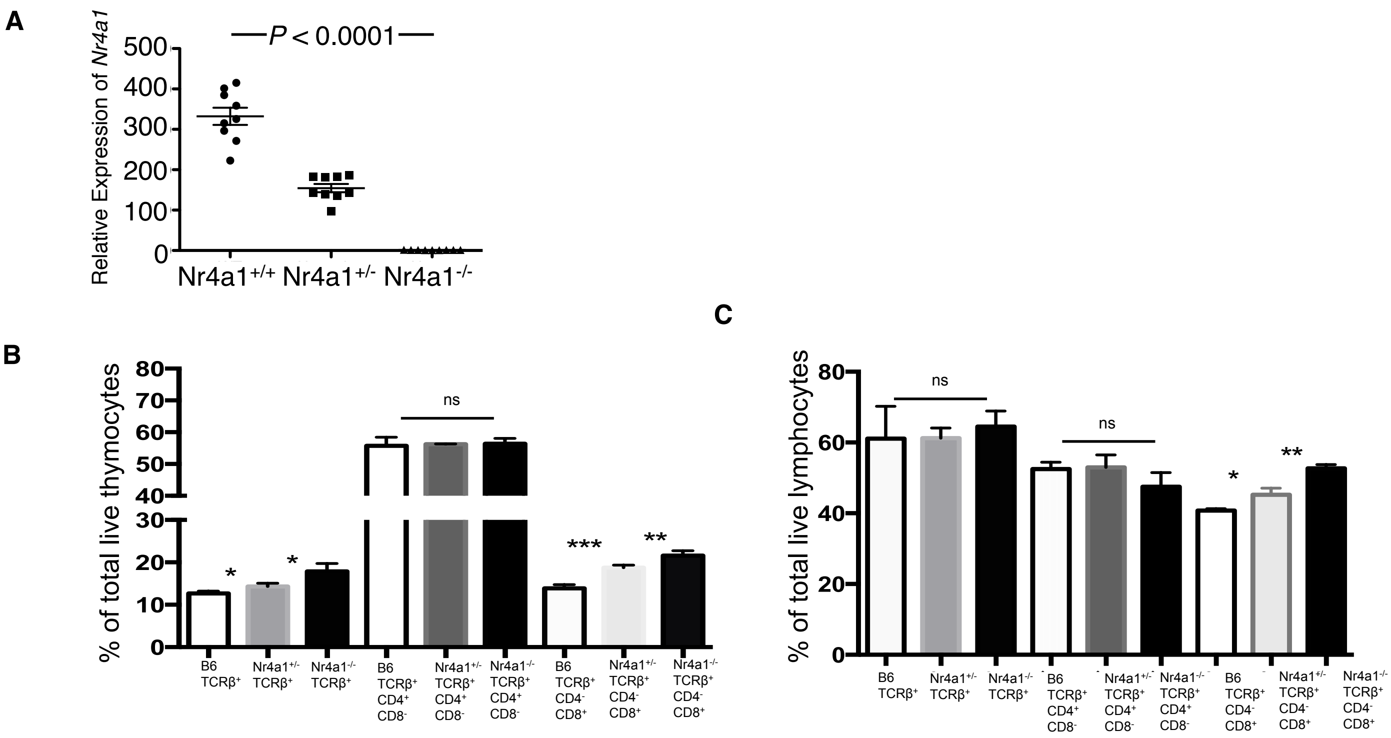

Supplemental Figure 3

The nuclear receptor Nr4a1 controls CD8 T cell development through transcriptional control of Runx3.

Heba N. Nowyhed, Tridu R. Huynh, Amy Blatchley, Runpei Wu, Graham D. Thomas and Catherine C. Hedrick

A

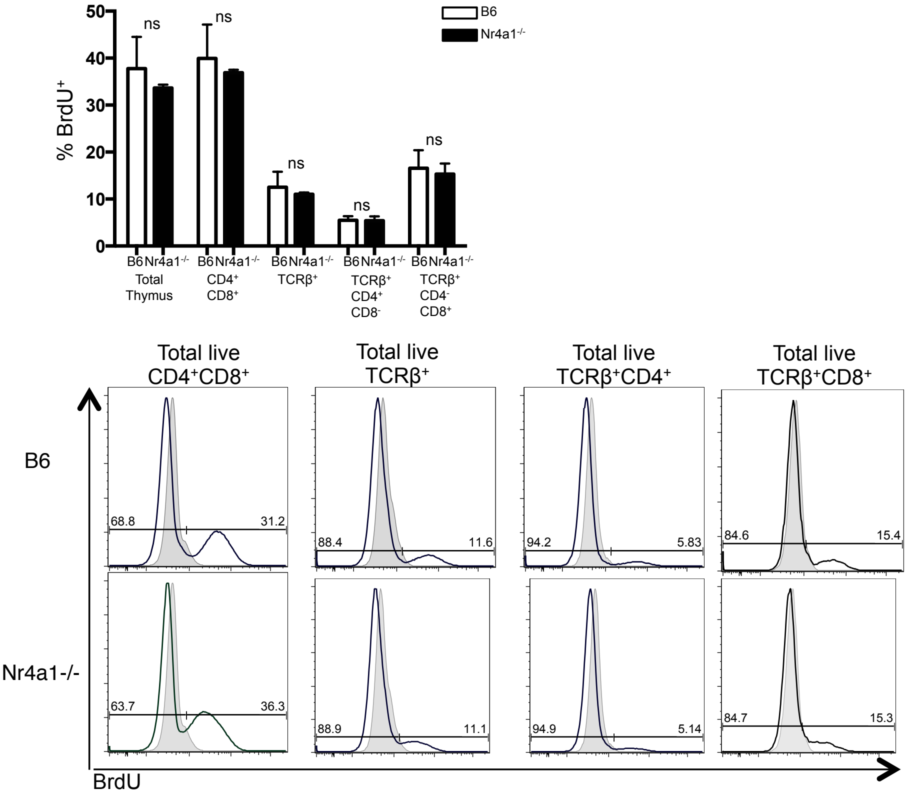

B

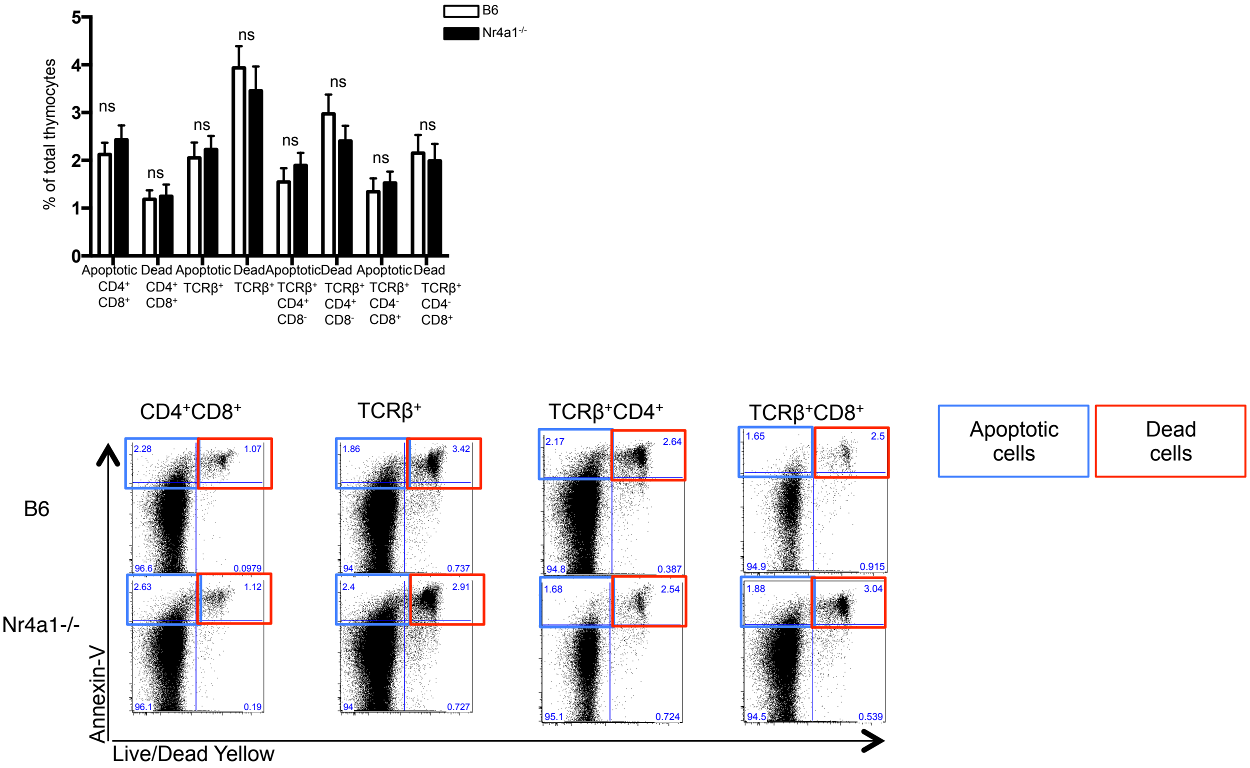

Supplemental Figure 4 *The nuclear receptor Nr4a1 controls CD8 T cell development through transcriptional control of Runx3.*

Heba N. Nowyhed, Tridu R. Huynh, Amy Blatchley, Runpei Wu, Graham D. Thomas and Catherine C. Hedrick

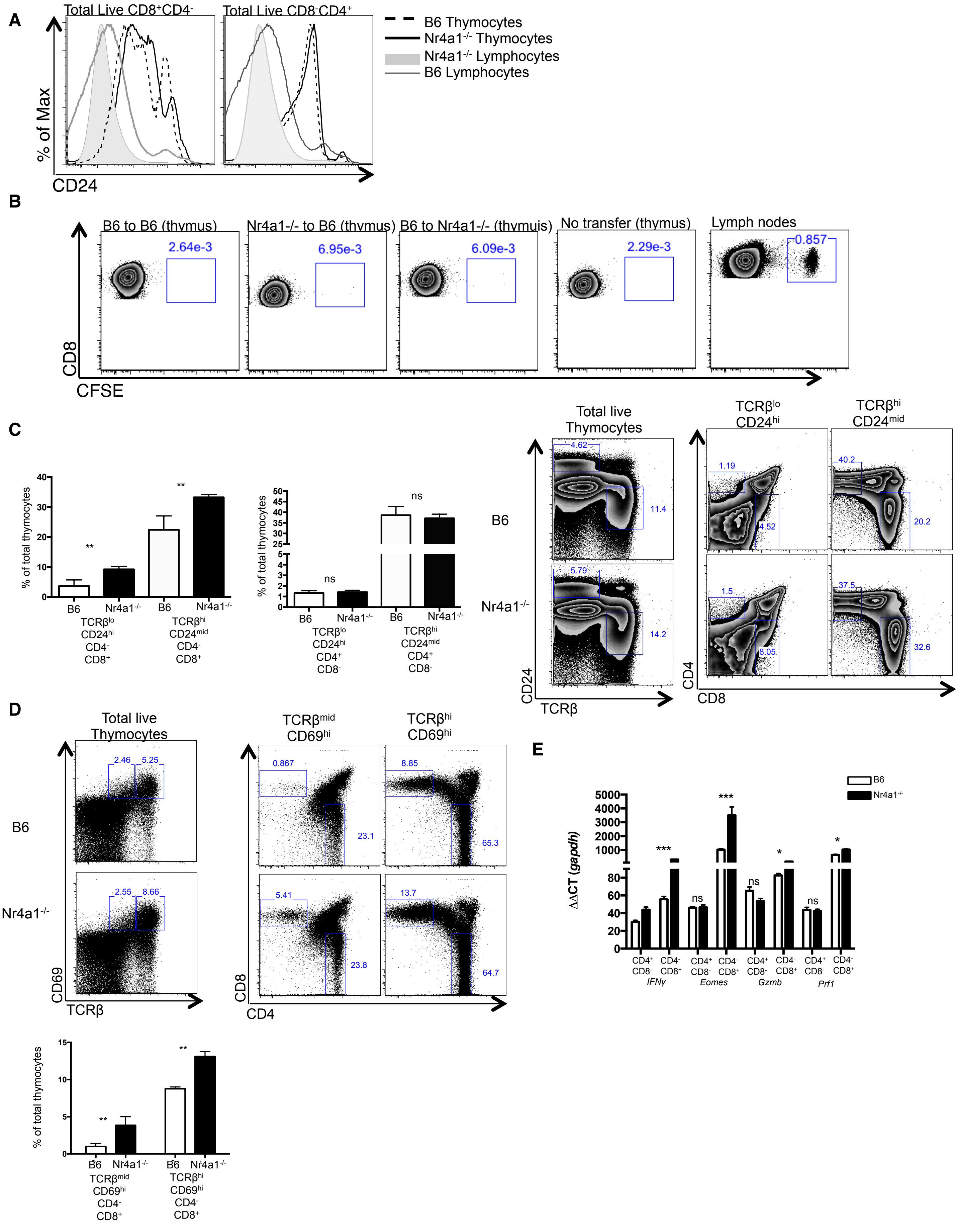

Supplemental Figure 5 *The nuclear receptor Nr4a1 controls CD8 T cell development through transcriptional control of Runx3.*

Heba N. Nowyhed, Tridu R. Huynh, Amy Blatchley, Runpei Wu, Graham D. Thomas and Catherine C. Hedrick

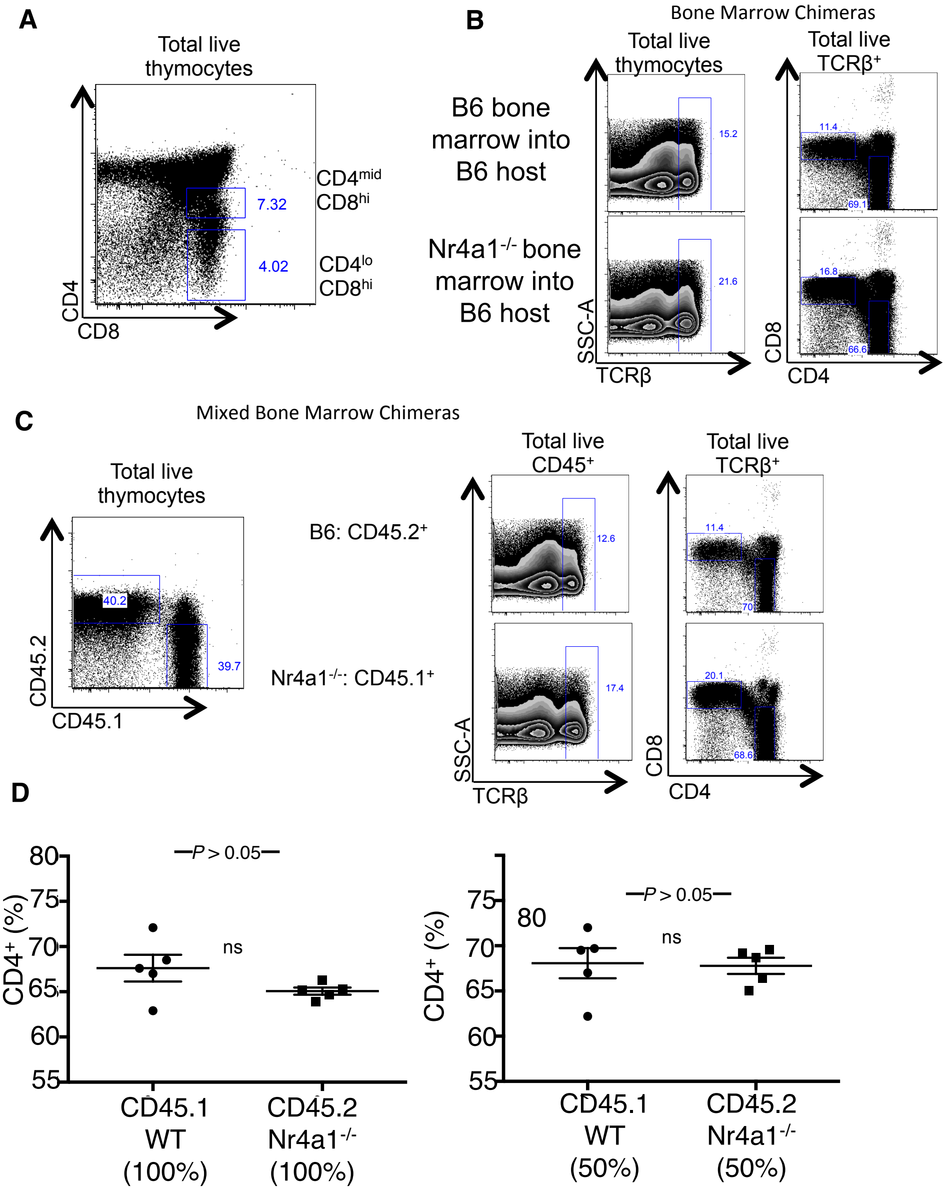

Supplemental Figure 6

The nuclear receptor Nr4a1 controls CD8 T cell development through transcriptional control of Runx3.

Heba N. Nowyhed, Tridu R. Huynh, Amy Blatchley, Runpei Wu, Graham D. Thomas and Catherine C. Hedrick

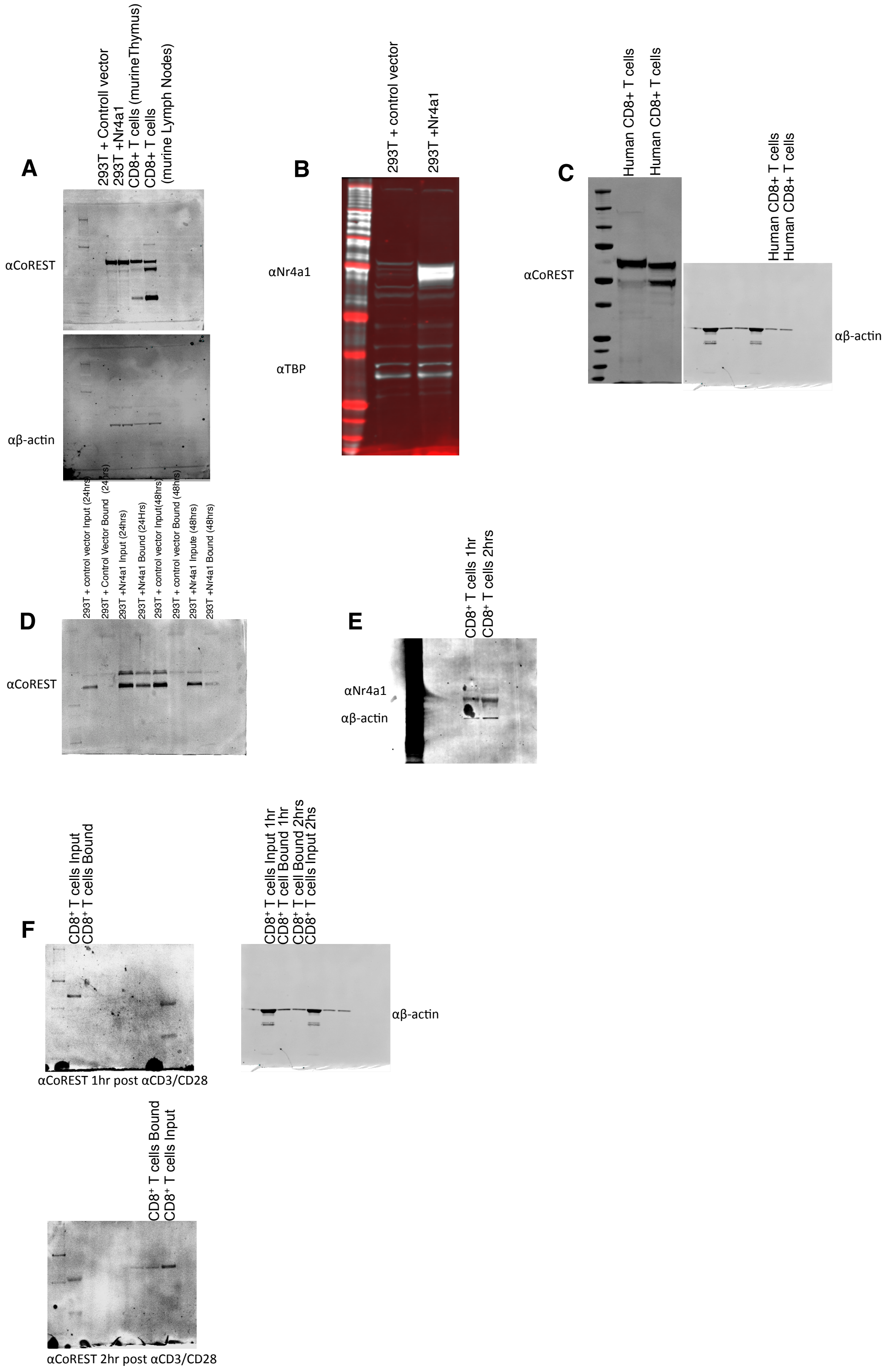

Supplementary Figure 1. Nr4a1 regulates thymic and peripheral $CD8^+$ T cell frequency. **(A)** Gated strategy for $CD4^-CD8^-$, $CD4^+CD8^+$, $CD4^+CD8^-$, and $CD4^-CD8^+$. Cells were first gated on total live cells. (**B**) Gating strategy for $CD44^+CD25^-$, $CD44^+CD25^+$, $CD44^-CD25^+$, $CD44^-CD25^-$. Cells were first gated on total live $CD4^-CD8^-$ thymocytes. (**C**) Gating strategy for total live $TCR\beta^+$ cells. Cells were first gated on total live thymocytes. (**D**) Gating strategy for $TCR\beta^+$ $CD4^-CD8^+$ and $TCR\beta^+$ $CD4^+CD8^-$. Cells were first gated on total live $TCR\beta^+$ thymocytes. (**E**) Gating strategy for $TCR\beta^+$ $CD69^{hi}$ cells. Cells were first gated on total live thymocytes. (**F**) Gating strategy of $CD4^+CD8^-$ and $CD4^-CD8^+$. Cells were first gated on total live $TCR\beta^+$ $CD69^{hi}$ cells. (**G**) Gating strategy of total live $TCR\beta^+$ cells. Cells were first gated on total lymphocytes. (**H**) Gating strategy of $CD4^+CD8^-$ and $CD4^-CD8^+$. Cells were first gated on total live $TCR\beta^+$ lymphocytes.

Supplemental Figure 2. The level of Nr4a1 expression effects the thymic development and peripheral frequency of $CD8^+$ T cells. **(A)** qPCR measuring the levels of *Nr4a1* RNA relative to *Gapdh* from total splenocytes of B6, $Nr4a1^{+/-}$, and $Nr4a1^{-/-}$ mice. (**B**) Frequency of total live $TCR\beta^+$, $TCR\beta^+$ $CD4^+CD8^-$, and $TCR\beta^+$ $CD4^-CD8^+$ in thymi of B6, $Nr4a1^{+/-}$, and $Nr4a1^{-/-}$ mice. (**C**) Frequency of total live $TCR\beta^+$, $TCR\beta^+$ $CD4^+CD8^-$, and $TCR\beta^+$ $CD4^-CD8^+$ in lymph nodes of B6, $Nr4a1^{+/-}$, and $Nr4a1^{-/-}$ mice. Data are representative of two separate experiments with at least three age and sex matched mice per group. Small

horizontal lines indicate the mean. *P* value, unpaired, two-tailed *t*-test. ns(p>0.05), *p<0.01, **p<0.001, ***p<0.0001.

Supplemental Figure 3. The absence of Nr4a1 does effect the rate of proliferation or apoptosis in thymocytes. (A) The frequency of BrdU incorporation (represented as %BrdU^{+}) in total live thymocytes, total live CD4^{+}CD8^{+}, total live TCRβ^{+}, total live TCRβ^{+}CD4^{+}CD8^{-}, and TCRβ^{+}CD4^{-}CD8^{+} thymocytes in B6 versus Nr4a1$^{-/-}$ mice. Histogram plots are representative BrdU incorporation in total live CD4^{+}CD8^{+}, total live TCRβ^{+}, total live TCRβ^{+}CD4^{+}CD8^{-}, and TCRβ^{+}CD4^{-}CD8^{+} thymocytes in B6 versus Nr4a1$^{-/-}$ mice. (**B**) Frequency of apoptotic (Annexin-V^{+}, Yellow^{-}) and dead (Annexin-V^{+}, Yellow^{+}) CD4^{+}CD8^{+}, TCRβ^{+}, TCRβ^{+}CD4^{+}CD8^{-}, and TCRβ^{+}CD4^{-}CD8^{+} thymocytes in B6 versus Nr4a1$^{-/-}$ mice. Dot plots show representative gating of apoptotic and dead cells in CD4^{+}CD8^{+}, TCRβ^{+}, TCRβ^{+}CD4^{+}CD8^{-}, and TCRβ^{+}CD4^{-}CD8^{+} thymocytes in B6 versus Nr4a1$^{-/-}$ mice. Data are representative of three separate experiments with at least three age and sex matched mice per group. Each symbol represents an individual mouse; small horizontal lines indicate the mean. *P* value, unpaired, two-tailed *t*-test. ns(p>0.05), *p<0.01, **p<0.001, ***p<0.0001.

Supplementary Figure 4. Nr4a1 controls the development of thymic CD8^{+} T cells. (A) Histogram plots representative of the level of surface CD24 on total live CD8^{+}CD4^{-} and CD8^{-}CD4^{+} from B6 thymocytes, Nr4a1$^{-/-}$ thymocytes, Nr4a1$^{-/-}$ lymphocytes, and B6 lymphocytes. (**B**) B6 and Nr4a1$^{-/-}$ CFSE labeled TCRβ^{+}

$CD8^+$ cells were adoptively transferred (via retro-orbital injection) into B6 or $Nr4a1^{-/-}$ mice, and the presence of the transferred cells in the thymus and lymph nodes was analyzed 10 days later. Numbers represent the percentage of total live $TCR\beta^+CD8^+CFSE^+$ cells at the time of analysis. (**C**) Frequency of $TCR\beta^{lo}CD24^{hi}CD4^-CD8^+$ and $TCR\beta^{hi}CD24^{mid}CD4^-CD8^+$ (left bar graph) and frequency of $TCR\beta^{lo}CD24^{hi}CD4^+CD8^-$ and $TCR\beta^{hi}CD24^{mid}CD4^+CD8^-$ (right bar graph) thymocytes in B6 versus $Nr4a1^{-/-}$ mice. Dot plots are representative gating strategies for $TCR\beta^{lo}CD24^{hi}$,$TCR\beta^{hi}CD24^{mid}$ cells and $CD4^-CD8^+$, $CD4^+CD8^-$ cells. (**D**) Frequency of $TCR\beta^{mid}CD69^{hi}$ and $TCR\beta^{hi}CD69^{hi}$ in B6 versus $Nr4a1^{-/-}$ thymi (Left dot plot column) and the frequency of $CD4^+CD8^-$ and $CD4^-CD8^+$ of $TCR\beta^{mid}CD69^{hi}$ thymocytes (middle dot plot column and bottom bar graph) and $TCR\beta^{hi}CD69^{hi}$ thymocytes (right dot plot column and bottom bar graph). Data are representative of three separate experiments with at least three age and sex matched mice per group. Small horizontal lines indicate the mean. *P* value, unpaired, two-tailed *t*-test. ns(p>0.05), *p<0.01, **p<0.001, ***p<0.0001. (**E**) qPCR measuring the levels of *IFNγ, Eomes, Gzmb, and Prf1* RNA relative to *Gapdh* from sorted $CD4^+CD8^-$ and $CD4^-CD8^+$ cells isolated from thymi of B6 versus $Nr4a1^{-/-}$ mice. Data are representative of three separate experiments with at least three age and sex matched mice per group. Small horizontal lines indicate the mean. *P* value, unpaired, two-tailed *t*-test. ns(p>0.05), *p<0.01, **p<0.001, ***p<0.0001.

Supplemental Figure 5. Nr4a1 intrinsically controls $CD8^+$ T cell development through Runx3 expression. (A) Gating strategy for total live

$CD4^{mid}CD8^{hi}$ and $CD4^{lo}CD8^{hi}$ thymocytes. (**B**) Gating strategy for $TCR\beta^{+}$ cells and $TCR\beta^{+}CD4^{+}$ and $TCR\beta^{+}CD8^{+}$ cells from thymi of bone marrow chimeras. (**C**) Gating strategy from 1:1 mixed bone marrow chimeras of total live CD45.1 and CD45.2 thymocytes. Dot plots representative of $CD45.1^{+}$ B6 cells and $CD45.2^{+}Nr4a1^{-/-}$ cells 10 weeks post reconstitution. **(D)** B6, $Nr4a1^{-/-}$, and B6:$Nr4a1^{-/-}$ 1:1 mixed bone marrow chimeric mice were analyzed for the frequency of $TCR\beta^{+}CD4^{+}$ cells in the thymus 10 weeks after reconstitution. Each symbol represents an individual mouse; small horizontal lines indicate the mean.

Supplemental Figure 6. Nr4a1 directly binds to CoREST (A) Western Blot analyses of CoREST expression in 293T cells transfected with an open reading frame, 293T cells transfected with Nr4a1, $TCR\beta^{+}CD8^{+}$ T cells isolated from B6 thymi, and $TCR\beta^{+}CD8^{+}$ T cells isolated from B6 lymph nodes. (**B**) Western Blot analyses of Nr4a1 expression in 293T cells transfected with a control vector, and 293T cells transfected with Nr4a1. (**C**) Western Blot analyses of CoREST expression from two different samples of $CD8^{+}$ T cells negatively isolated from whole human blood. (**D**) Analyses of the interaction of Nr4a1 and CoREST. Co-IP was performed with anti-Nr4a1 antibody and western blots were developed with anti-CoREST antibody. (**E**) Western Blot analyses of Nr4a1 expression in $CD8^{+}$ T cells isolated from human blood and stimulated *in vitro* with αCD3αCD28 dynabeads for 1hr and 2hrs. (**F**) Analyses of the interaction of Nr4a1 and CoREST in human $CD8^{+}$ T cells isolated from the blood and stimulated *in vitro* with αCD3αCD28 dynabeads for 1hr and 2hrs. Co-IP was performed with anti-

Nr4a1 antibody and western blots were developed with anti-CoREST antibody.

Data are representative of two separate experiments.
